# Effect of short dentin etching and water storage on bonding of resin composite to dentin with universal and two-step self-etch adhesive systems

**DOI:** 10.1038/s41598-025-31027-9

**Published:** 2025-12-15

**Authors:** Basma Ahmed, Omar Abd El-Maksoud, Ramy Ahmed Wafaie, Salah Hasab Mahmoud

**Affiliations:** 1 Conservative Dentistry Department, Faculty of Oral and Dental Medicine, Delta University for Science and Technology, Gamasa, 35712 Egypt; 2 Operative Dentistry Department, Faculty of Dentistry, Mansoura University, Mansoura, Egypt

**Keywords:** Bond strength, Short dentin etching, Self-etch adhesive, Universal adhesive, Water storage, Health care, Materials science, Medical research

## Abstract

To evaluate the effect of short dentin etching and 6-month water storage on the microtensile bond strength (µTBS) of universal and two-step self-etch adhesives to dentin. Mid-coronal dentin specimens obtained from 56 third molars were assigned into two groups according to the adhesive type (*n* = 28); universal adhesive (Scotchbond Universal Plus Adhesive, 3 M Oral Care) and two-step self-etch adhesive (Clearfil SE Bond, Kuraray Noritake). Each group was further divided into two subgroups (*n* = 14) based on the application protocol: self-etch (SE) and etch-and-rinse with short dentin etching (E&R/SDE) for 3 s. After bonding and composite application, half of the specimens were stored in 37 ± 1 °C distilled water for 24 h (immediate), while the other half were stored for 6 months (aged). Thereafter, specimens were cut into 1 mm² beams using a slow-speed diamond saw under copious water cooling. The beams were then subjected to a tensile force at a cross-head speed of 0.5 mm/min in a universal testing machine until failure occurred. The µTBS was then calculated in megapascals (MPa) by dividing the load at failure by the cross-sectional area of each beam. The values of five beams were averaged to obtain one µTBS value per tooth, and accordingly, data were analyzed using three-way ANOVA and Tukey HSD post-hoc tests (*p* < 0.05). Failure modes were recorded. The application protocol and the storage time significantly influenced the µTBS results (*p* < 0.001). Regarding the application protocol, E&R/SDE for 3 s yielded significantly higher bond strength values than the SE for both adhesives (*p* < 0.05). In terms of storage-time, all groups exhibited a statistically significant reduction in bond strength after 6-month water storage (*p* < 0.05). On the contrary, no statistically significant difference was detected between the two adhesives irrespective of the application protocol or the storage time (*p* > 0.05). The predominant failure mode observed for immediate groups was mixed failure, while adhesive failure was the most frequently noted after 6 months. Despite the beneficial effect of E&R/SDE for 3 s in improving the bond strength of universal and 2-step self-etch adhesives to dentin, the 6-month water storage negatively affected the bonding performance of both adhesives.

**Clinical relevance:** E&R/SDE enhanced μTBS at both storage times and may contribute to better bond stability, although all groups exhibited degradation after 6-month water storage, which necessitates further clinical validation.

## Introduction

Dental adhesion has added remarkable changes and improvements to the field of restorative dentistry through achieving satisfactory clinical performance along with preserving the tooth structure^1^. Despite continuous progress in adhesive technology, maintaining the long-term durability of resin–dentin bonding remains a significant challenge. This critical process relies on forming a stable hybrid layer through adequate resin infiltration into the scaffold produced by demineralizing the dentin collagen matrix^2,3^. The key to achieving successful restorative outcomes relies on proper hybridization, which allows the resin-dentin interface to withstand various oral challenges including masticatory forces and hydrolytic degradation^4^. Previous studies^5–8^ have reported a significant decline in the resin-dentin bond strength owing to the heterogenicity and hydrophilicity of dentin, the activity of dentinal matrix enzymes, and the complex interaction between the adhesive and the tooth structure.

Adhesive systems were classified mainly into etch-and-rinse (E&R) and self-etch (SE) adhesives based on their adhesion strategy^9^. For many years, E&R adhesives were the most widely used by clinicians. However, the higher incidence of post-operative sensitivity, incomplete resin infiltration into the full depth of demineralization, and a relatively complex multi-step application have contributed to their gradual phase-down^10,11^. In response, SE adhesives were developed with the aim of simplifying enamel and dentin bonding and reducing the overall clinical application time^12,13^.

SE adhesives do not require separate etching and rinsing steps which eliminate the risk of over-etching, over-wetting and over-drying of dentin, thereby, decreasing the clinical procedural errors^14–16^. They contain acidic monomers which have the ability to etch and prime enamel and dentin simultaneously^17^. These monomers partially demineralize the underlying tooth substrate, whereas the smear layer and mineral phase are not completely removed. Rather, the acidic monomers penetrate the tissue through the porosities of the smear layer and the superficial dentin^10,18,19^. The interaction of the monomers with both the collagen phase of dentin as well as hydroxyapatite mineral phase^19,20^ contributes to an enhanced cohesion of the infiltrated resin after polymerization and probably to better resistance to hydrolysis^21^. Irrespective of the etching aggressiveness of the incorporated acidic monomers, a crucial shortcoming of SE adhesives is their inability to etch enamel adequately, resulting in a shallow and poorly defined enamel etching pattern^10^. Since a strong enamel bond is essential for establishing an effective marginal seal and for protecting the more vulnerable dentin bond from degradation^22^, considerable attention has been directed toward overcoming this limitation by incorporating a separate acid-etching step for enamel only, a technique referred to as selective enamel etching^10^.

SE adhesives are categorized into two groups based on the number of clinical application steps. Two-step self-etch adhesives (2-SEAs) are considered the gold standard adhesives involving initial application of hydrophilic acidic primer followed by hydrophobic bonding resin. This renders the interface more hydrophobic ensuring long-term bond durability and preventing the degradation of the adhesive interface^10,23,24^. These adhesives exhibited good clinical performance to dentin as reported by previous studies^25–27^. One- step self-etch adhesives (1-SEAs) are more simplified and easier in application; however, they might not provide effective dentin bonding due to their excessive hydrophilicity that could lead to bond degradation over time, in addition to the potential phase-separation between hydrophilic and hydrophobic components^28–30^.

Despite the well-recognized drawbacks of 1-SEAs, their exceptional user-friendliness has continued to attract clinicians worldwide. Consequently, substantial efforts have been devoted to developing new adhesives that offer enhanced properties and more predictable clinical performance^31^. These advancements have led to the emergence of more versatile adhesives, known as “universal adhesives” or “multi-mode adhesives”, which were designed to combine the simplicity of self-etch systems with the flexibility of different bonding strategies^32,33^. Universal adhesives have become increasingly popular and widely adopted in restorative dentistry in recent years, as they can be used in E&R, SE, and SEE modes. The versatility of these adhesives enables them to be used with different substrates according to the clinical situation^10,34–36^. This evolution was mainly attributed to the inclusion of acidic functional monomers in their composition, such as 10-methacryloxydecyl dihydrogen phosphate (10-MDP). This molecule forms stable ionic bond to calcium through a nanolayered structure of MDP-Ca salts which maintains the collagen fibrils protected, reduces water sorption, and enhances the durability of bonding. Also, the combination of various monomers in their matrix stabilizes the bond between the hydrophilic tooth structure and hydrophobic resin-based restorative materials^37–41^.

Phosphoric acid etching plays an important role in the adhesion process through dissolving the smear layer and demineralizing the tooth structure^42^. In an effort to improve the bonding efficiency of adhesives used in SE mode to dentin, a new off-label technique known as short dentin etching (SDE) has emerged. This involves applying low-concentration phosphoric acid (32%) for only 3 s on the dentin surface, followed by 5 s rinsing and gentle air-drying. Based on previous literature^43–45^, this protocol may hypothetically enhance bonding durability, a benefit that could be related to the potential of SDE to produce a moderately demineralized dentin surface while preserving high calcium content within the hybrid layer. By such approach, improved dentin bonding performance could be achieved without unnecessarily extending the application time.

There is debate regarding the actual suitability and reliability of performing SDE for 3 s with adhesives used in the SE mode. To date, and to the best of the authors’ knowledge, studies evaluating and comparing the effect of this off-label protocol on the dentin bond strength of Universal adhesives and the gold-standard 2-SEA are extremely lacking. Furthermore, the impact of water storage when this proposed approach is applied with both adhesives has not yet been investigated in the literature. Therefore, this in vitro study aimed to evaluate the effect of E&R/SDE for 3s on the µTBS of universal and 2-SEA adhesives to dentin after water storage for 24 h (immediate) and 6 months (aged). The null hypothesis tested was that there would be an absence of differences in bond strength to dentin regardless of the adhesive type, the application protocol, and the water storage time.

## Materials and methods

### Materials

A universal adhesive (Scotchbond Universal Plus Adhesive, 3 M Oral Care, St. Paul, MN, USA) and a two-step self-etch adhesive (Clearfil SE, Kuraray Noritake Dental Inc., Okayma, Japan) were employed in this study. Both adhesives were used in conjunction with a nanohybird resin composite (Neo Spectra ST LV, Dentsply Sirona GmbH, Konstanz, Germany). The full description of the materials used and their handling procedures are presented in Table 1. Photo-polymerization was performed using a light emitting diode (LED) curing unit (Elipar S10, 3 M Oral Care) with a wave length of 430–480 nm and a power intensity of 1200 mW/cm2 after being inspected by built-in radiometer.

### Teeth selection

The current study was approved by the Research Ethics Committee of Faculty of Oral and Dental Medicine, Delta University for Science and technology, Gamasa, Egypt under protocol number (DU:025021304). All methods were carried out in accordance with relevant guidelines and regulations of Helsinki Declarations. Sound freshly human third molars were extracted for therapeutic reasons unrelated to the study, with prior informed consent from healthy individuals who were seeking dental care at Oral Surgery Department Clinic. The patients were voluntary donating the extracted teeth to the faculty for utilizing in research purpose. The selected teeth were inspected for being free from caries and cracks using a stereomicroscope (SZ TP, Olympus, Tokyo, Japan) under 20x magnification. All teeth exhibited comparable dimensions mesiodistally (9 ± 0.5 mm) and buccolingually (9.5 ± 0.5 mm). The teeth cleaning process started with removing any soft or hard tissue remnants using a hand scaler (Zeffiro, Lascod, Florence, Italy), then the teeth were rinsed under running water. Subsequently, the cleaned teeth were kept in 0.5% chloramine T solution for 2 days, then stored in distilled water at 37 ± 1 °C using an incubator (BTC, BioTech Company, Cairo, Egypt until being used^46–48^.

### Sample size calculation

Sample size was calculated using a statistical software program (G*Power, Ver.3.1.9.1, Dusseldorf, Germany) based on a previous study^49^. A two-tailed test with an effect size of 0.92, a significance level (α) of 0.05, and 80% statistical power were considered. Therefore, a representative sample composed of 7 teeth per subgroup was selected for this study.

### Study design

**Fig. 1:**
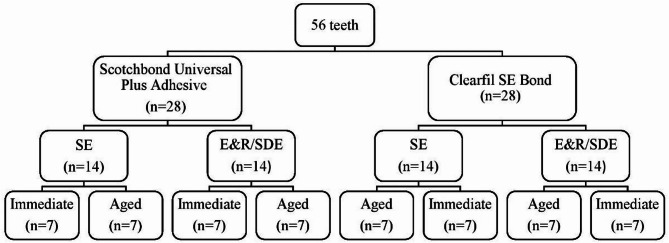
A schematic diagram showing the study design and the grouping system.

The experimental design was composed of three study factors defined as: (i) the adhesive type at two levels (Scotchbond Universal Plus and Clearfil SE Bond); (ii) the application protocol at two levels (SE and E&R/SDE for 3 s); (iii) the water storage time at two levels (24 h and 6 months). The present study evaluated µTBS to dentin and the different failure modes. A schematic illustration for the grouping system of the specimens is presented in Fig. 1.

### Specimen preparation

The roots of molar teeth were embedded vertically in self-curing acrylic blocks up to 2 mm below the cementoenamel junction (C.E.J). For each tooth, the occlusal one third of the crown was sectioned parallel to the occlusal table using a slow-speed diamond saw (Isomet 4000, Buehler Ltd., Lake Bluff, Illinois, USA) under copious water coolant system in order to obtain flat mid-coronal dentin surface. The stereomicroscope was used to verify that the dentin surfaces were free from any remaining enamel. In order to create a standardized smear layer for all specimens, the exposed dentin surfaces were wet-ground with 600-grit silicon carbide paper (Microcut, Buehler Ltd.) for 60 s under continuous water flow^13,44,50^.

### Restorative procedures

Regarding Scotchbond Universal Plus adhesive, the material was applied in SE mode following the manufacturer’s Instructions for Use (IFU). Briefly, the adhesive was applied with active application using a disposable applicator for 20 s. Subsequently, a gentle stream of air was applied for a minimum of 5 s until a shiny film appeared and no longer moved in the air stream. Finally, the adhesive layer was light-cured for 10 s using a LED curing unit. According to the original manufacturer’s IFU, the conventional E&R bonding protocol recommends that, prior to applying the adhesive, phosphoric acid etching gel (30–40%) is applied to the prepared tooth structure and allowed to react for 15 s, rinsed thoroughly with water, and dried with water-free and oil-free air. Deviating from these standard instructions, the experimental off-label protocol involved applying 32% phosphoric acid etchant (Uni-etch, Bisco, Inc., Schaumburg, IL, USA) to the dentin surface for only 3 s. After SDE, rinsing, and air-drying, the adhesive was applied as in SE mode.

For Clearfil SE Bond, the adhesive was applied in SE mode following the manufacturer’s IFU. The primer was applied with a disposable brush tip and left in place for 20 s. After conditioning the tooth surface, a mild oil-free air stream was used to evaporate the volatile ingredients. The adhesive was then applied to the entire surface with a disposable brush tip followed by a gentle oil-free air stream to create a uniform adhesive film. The adhesive was subsequently light-cured for 10 s. No E&R protocol is reported by the manufacturer of Clearfil SE and it was utilized in the off-label protocol using E&R/SDE for 3 s following the same steps adopted for Scotchbond Universal Plus Adhesive to ensure standardization. All the bonding procedures were described in full details in Table 1.

A custom-made rounded Teflon mold (6 mm x 6 mm and 4 mm in height) was used for resin composite build-up. A 4 mm thick layer of nanohybird resin composite (Neo Spectra ST LV) was applied in 2 horizontal increments of 2 mm each using a titanium-coated hand instrument (curved paddle “LRT,” Nordent, Bonnie Lane, USA). Each composite increment was light-cured separately from the occlusal surface following the manufacturer’s instructions. The Teflon mold was then removed and an additional curing was performed for 20 s to all sides of the specimen to ensure adequate polymerization of the resin composite restoration^49,51,52^. All procedures were performed by a single operator. Half of the restored specimens in each subgroup were stored in distilled water at 37 ± 1 °C for 24 h (immediate), while the other half was stored for 6 months (aged)^53^.

### Test procedure

Each specimen was longitudinally sectioned in mesio-distal and buccal-lingual directions perpendicular to the adhesive interface producing resin-dentin beams with cross sectional area of approximately 1 mm² using a slow-speed diamond saw (Isomet 4000) under copious water cooling^49,54,55^. Five beams for each specimen were randomly selected for testing following previously conducted studies^49,55^. For more standardized measurements, only central beams were chosen to be tested while peripheral ones were excluded^54^.

Each beam was attached separately to a Geraldeli’s jig using a cyanoacrylate adhesive (Zapit, DVA Inc, Corona, CA, USA), then subjected to tensile force delivered gradually from a universal testing machine (Instron, Model: 3345, Nor-wood, MA, USA) at cross-head speed of 0.5 mm/min until failure occurred to obtain the maximum load (P) in Newton (N). A digital caliper was used to measure the cross-sectional area (CA) in mm^2^ of each beam with 0.01 mm accuracy. The µTBS was then calculated in mega-Pascal (MPa) using the Bluehill Lite software (Instron, Norwood) by dividing the load at failure by the cross-sectional area. The values of five beams were averaged to obtain one µTBS value per tooth, and accordingly, the tooth was considered the statistical unit^34,56^.

After testing, beams were examined from both sides under a stereomicroscope at 40x magnification to assess the modes of failure which were categorized as follows: adhesive failure at the resin-dentin interface, cohesive failure within dentin or resin composite, and mixed failure involving a combination of adhesive and cohesive failures^34,44^.

**Table 1:** Composition and protocol of adhesive systems.

Material	Type	Manufacturer	Composition	Application technique	Batch No.
Scotchbond Universal Plus Adhesive	Universal adhesive	3 M Oral Care	HEMA,10-MDP, Vitrebond copolymer, ethanol, water, silane, CQ-based initiator, BPA-free dimethacrylate resin	In SE (manufacturer protocol): 1-Apply with active application for 20s. 2-Air dry for 5s. 3-Light-cure for 10s. In E&R/SDE (experimental off-label protocol): 1-Apply etchant for 3s. 2-Rinse thoroughly with stream of water. 3-Gently apply air for 10s. 4-Apply the bond as in SE.	10445962
Clearfil SE Bond	Two-step self-etch adhesive	Kuraray Noritake	Primer: MDP, HEMA, hydrophilic dimethacrylate monomer, CQ, water, catalyst. Bond: 10-MDP, bis-GMA, HEMA, CQ, hydrophobic dimethacrylate monomer, microfiller, catalyst.	In SE (manufacturer protocol): 1-Apply the primer and leave for 20s. 2-Dry thoroughly with mild air flow. 3-Apply the bond. 4-Air flow gently. 5-Light-cure for 10s. In E&R/SDE (experimental off-label protocol): 1-Apply etchant for 3s. 2-Rinse thoroughly with a stream of water. 3-Gently apply air for 10s. 4-Apply the primer and bond as in SE.	000202
Neo Spectra ST LV	Nano-hybrid composite	Dentsply Sirona	Spherical pre-polymerized SphereTEC fillers, non-agglomerated barium glass and ytterbium fluoride, highly dispersed methacrylic polysiloxane nano-particles.		2305000755

### Statistical analysis

The obtained data were analyzed using the Statistical Package for the Social Sciences (IBM-SPSS, version 24, Armonk, NY, USA). Since data revealed normal distribution according to Kolmogorov-Smirnov test (*p* > 0.05) and the equality of variances was confirmed by Levene test (*p* > 0.05), a parametric statistical procedure was used to test the significance of difference between group variability. A three-way analysis of variance (ANOVA) was used to statistically analyze the effect of different variables on the bond strength to dentin. Additionally, The Tukey HSD post-hoc multiple comparison test was used to test the significance of difference between groups. The significance level was set at p˂0.05 for all analyses.

## Results

The mean bond strength values (µTBS) and the standard deviation for each group are displayed in Table 2. The 3-way ANOVA test revealed that adhesive type had no significant effect on bond strength values (*p* = 0.06), while the application protocol and the storage time showed a significant effect on the results (*p* < 0.001).

**Table 2:** Means and standard deviations of µTBS values in MPa among tested adhesives.

Groups	Immediate	Aged
Scotchbond Universal Plus (SE)	41.29 ± 4.092^B^	25.97 ± 3.440^C^
Scotchbond Universal Plus (E&R/SDE)	56.92 ± 5.375^A^	38.08 ± 5.851^B^
Clearfil SE (SE)	45.20 ± 5.702^B^	28.47 ± 5.031^C^
Clearfil SE (E&R/SDE)	59.87 ± 5.287^A^	39.18 ± 4.583^B^

*Different letters indicate statistically significant differences at a level of significance (*p* < 0.05).

For immediately tested groups, the results of Tukey HSD multiple comparison test revealed no statistically significant difference between Scotchbond Universal Plus adhesives and Clearfil SE either applied in SE protocol (*p* = 0.819) or in E&R/SDE protocol (*p* = 0.952). However, significantly higher bond strength values were obtained when dentin was etched for both adhesive materials in comparison to non-etched groups (*p* < 0.05). For aged groups, no statistically significant difference was found between both adhesives in SE protocol (*p* = 0.980) and E&R/SDE protocol (*p* = 0.999). Nevertheless, dentin etching has significantly improved the bond strength values when compared to non-etched groups (*p* < 0.05). After 6-month water storage, all groups showed a significant decrease in dentin bond strength when compared to immediate groups (*p* < 0.05).

**Fig. 2:**
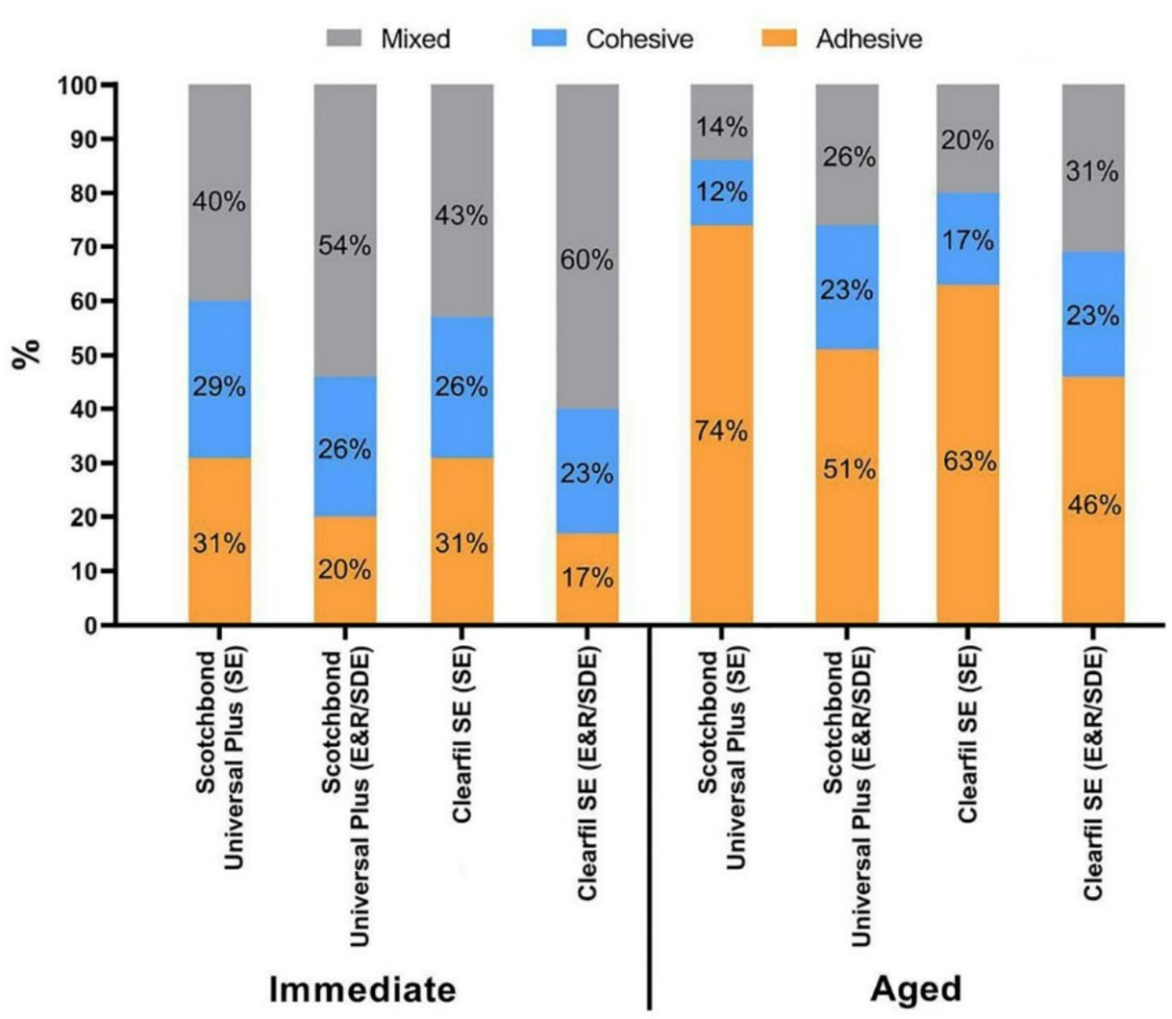
Percentage values of failure patterns for tested groups.

**Fig. 3:**
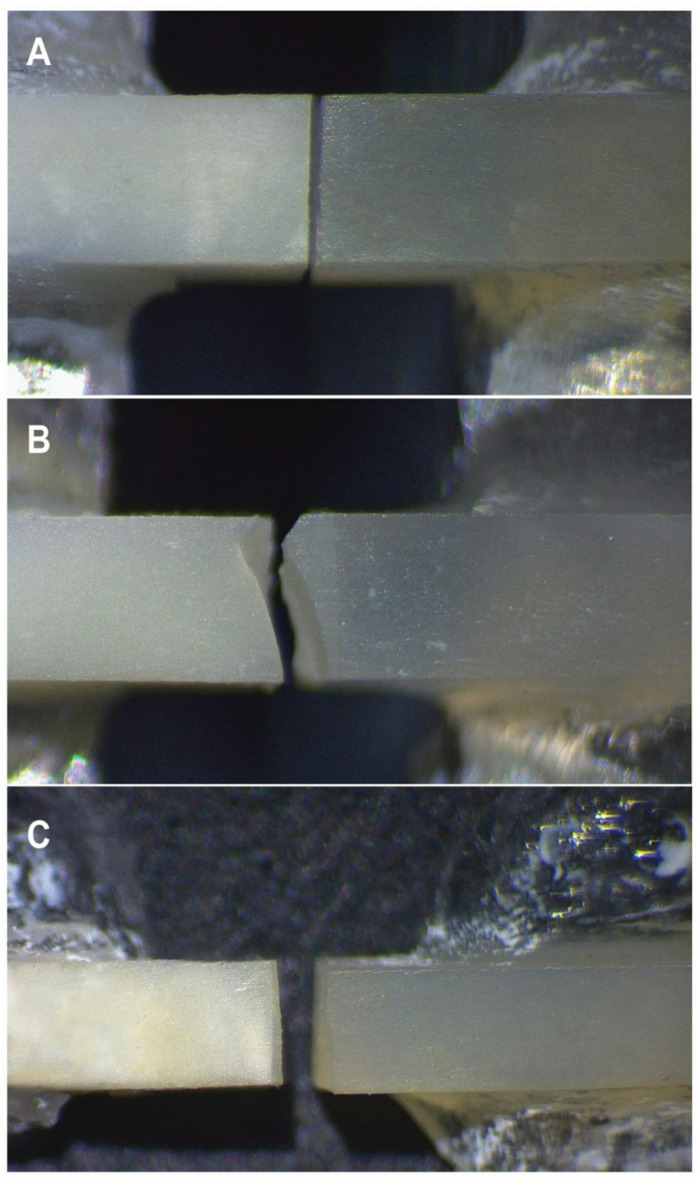
A representative photograph showing: (**A**) adhesive failure, (**B**) cohesive failure, (**C**) Mixed failure.

The percentage values of failure patterns for all groups are presented in Fig. 2. The predominant failure mode observed for immediate groups was mixed failure regardless the adhesive type or application protocol. In aged groups, the most frequent failure mode observed was adhesive failure. Representative images of different failure modes are shown in Fig. 3.

## Discussion

The primary goal for contemporary adhesive dentistry is to achieve firm and durable bonding between the restorative material and tooth structure. The success of any dental adhesive is based mainly on its ability to bond effectively to enamel and dentin. However, bonding to dentin remains a major challenge due to its heterogeneous composition, complex histology, and intrinsic moisture. Additionally, the mismatch in the nature between the hydrophilic dentin and hydrophobic adhesive material has a great impact on the dentin-bonding performance^30,43^.

As clinicians certainly prefer less complicated and more versatile adhesive materials, the newly launched Scotchbond Universal Plus Adhesive was selected to be used in this study being a novel adhesive material offering several advantages over its predecessor; Scotchbond Universal Adhesive, including a higher degree of conversion, improved in situ polymerization, greater mechanical strength, and lower viscosity, which facilitates better infiltration into etched dentin^36^. Moreover, Clearfil SE was chosen to be the comparative counterpart as it is still the reference and the gold standard SE adhesive material due to its higher bond strength to dentin and excellent clinical outcomes^10,26^. Both adhesives fell within an approximate mild-acidity range to minimize variability. Mild SE adhesives partially remove the smear layer creating a thin hybrid layer by demineralizing dentin only to a depth of 1 μm leaving substantial amount of hydroxyapatite attached to collagen fibrils to form a chemical bond with carboxylic or phosphate groups of functional monomers^9,23^.

SDE technique using 32% phosphoric acid was investigated in the present study being a promising experimental protocol that combines the simplicity associated with SE adhesives with the potential bonding advantages of dentin-etching step for 3 s, without losing most minerals essential for effective bonding, unnecessarily increasing the clinical application time or encountering the known drawbacks of the conventional E&R protocol for 15 s^44,45^. Water storage is considered one of the ideal and simplified aging methods for evaluating the bonding performance of adhesive materials. Thus, half of the specimens were subjected to 6-month storage in distilled water to duplicate the challenges that occurs in the oral cavity^39,41^.

Although the findings of the present study showed no differences attributable to the adhesive type, both the application protocol and the time of water storage induced significant difference on the µTBS results. Therefore, the null hypothesis was rejected. Both adhesives showed higher µTBS with E&R/SDE compared to SE in both immediate and aged groups. This could be related to the removal of smear layer without overexposing the demineralized collagen fibrils or decreasing the calcium content. This could improve the chemical interaction between 10-MDP and dentin resulting in higher bonding durability. While, using these adhesives in SE mode prevented the optimum bonding with dentin due to the presence of smear layer^3,34^. This outcome is supported by the result of a previous study^4^ which revealed that a separate etching step for dentin was necessary for mild adhesives in order to increase dentin bonding effectiveness. A previous finding^44^ reported that micromechanical and chemical bonding mechanisms were enhanced after SDE resulting in 30% significant increase in bond strength when compared to SE mode.

The 6-month water storage resulted in a significant decline in the bond strength of both adhesives. This could be ascribed to water sorption into adhesive layer leading to hydrolysis and subsequent breakdown of the collagen matrix and polymeric chains. The compromised hybrid layer as well the dissolution of resin-dentin interface could weaken the bond strength over time^4,48^. This is in congruence with a previous study^44^ which reported a significant deterioration of the bond strength after water storage. However, a conflicting report^53^ indicated that µTBS did not significantly change following 6 months of water storage. This contradiction may stem from the different adhesive categories examined, as well as the use of bovine incisors rather than human molars. The underperformance of both adhesives in SE mode after water aging indicates higher interfacial susceptibility to hydrolysis compared to E&R/SDE. This could be related to the presence of residual smear layer which might hinder adequate monomer infiltration into dentin adversely affecting long-term resin-dentin degradation^4^.

Scotchbond Universal Plus adhesive demonstrated dentin bond strength comparable to that of Clearfil SE Bond under both evaluated conditions, despite the two adhesives being classified into different categories and varying in their compositions. This might be attributed to modifications in the chemical composition of Scotchbond Universal Plus, as well as the presence of polyalkenoic acid copolymer, which likely contributes to a behavior similar to the gold-standard Clearfil SE Bond through promoting chemical interaction with calcium in hydroxyapatite, thereby enhancing bond strength^43,50^. Furthermore, the incorporation of the 10-MDP functional monomer in both adhesives underlies their consistent bonding performance. This monomer is mainly responsible for the formation of water-insoluble calcium salts which protect the hybrid layer from degradation and contribute to the stability of adhesive interface^16,52^. This outcome is supported by a previous report^35^ which stated that both universal and 2-SEA adhesives provided similar bonding efficacy to dentin. However, another study^17^ encouraged the use of 2-SEA adhesives over certain universal adhesives. A possible explanation for this discrepancy is the variation in adhesive types tested and the evaluation methods employed.

The study results showed high incidence of mixed failure pattern for all immediate groups with substantial increase in percentage of adhesive failure when these adhesives used in SE mode. This could be explained by the higher bond strength observed for both adhesives with the E&R/SDE step^34^. This is consistent with a previous study^36^ that reported an increase in non-adhesive failure due to the enhanced bonding ability. Conversely, the most frequent failure mode noted for all groups after 6-month water storage was the adhesive failure. This could likely be a consequence of the reduction in bond strength over time due to the hydrolytic degeneration of adhesive system as a result of slow water hydrolysis promoting adhesive breakdown^47^.

Although SDE improved immediate bonding, the long-term degradation after aging highlights the need for caution when extrapolating to clinical situations. Moreover, certain limitations of this in-vitro study further underscore this concern. Only a 3-second etching time for dentin was employed, indicating that different etching times should be investigated to provide a more comprehensive evaluation. Additionally, the 6-month storage period may not fully capture the effects of long-term aging. The specimens were subjected to tensile loading to induce fracture without prior thermal and/or mechanical cycling. Furthermore, the absence of 15 s E&R control, along with the limited number of adhesives tested, emphasizes the need for further research examining a broader range of adhesive systems relative to a standard control. The in-vitro nature of the study may also not fully replicate clinical conditions, including temperature fluctuations and thermal changes. The smear layer obtained using 600-grit SiC paper is thinner than that typically formed clinically with diamond burs. Considering these factors, future clinical studies are warranted to validate these preliminary findings.

## Conclusions

Within the limitations of this in-vitro study, the findings suggest while the experimental E&R/SDE for 3 s may improve the immediate bonding performance of both universal and 2-SEA adhesives, the deterioration observed after 6 months of water storage underscores that long-term bond durability remains a significant concern.

## Data Availability

The datasets used and/or analysed during the current study are available from the corresponding author on reasonable request.
